# Transgenic RXLR Effector *PITG_15718.2* Suppresses Immunity and Reduces Vegetative Growth in Potato

**DOI:** 10.3390/ijms20123031

**Published:** 2019-06-21

**Authors:** Jiao Wang, Cungang Gao, Long Li, Weilin Cao, Ran Dong, Xinhua Ding, Changxiang Zhu, Zhaohui Chu

**Affiliations:** 1 State Key Laboratory of Crop Biology, Shandong Agricultural University, Tai’an 271018, China; jiaosunshine89@126.com (J.W.); 18763825797@163.com (C.G.); CWL3237@126.com (W.C.); xhding@sdau.edu.cn (X.D.); zhchx@sdau.edu.cn (C.Z.); 2 Shandong Provincial Key Laboratory of Vegetable Disease and Insect Pests, College of Plant Protection, Shandong Agricultural University, Tai’an 271018, China; dong_ran02@163.com; 3 College of Agronomy, Shandong Agricultural University, Tai’an 271018, China; lilong130682@163.com; 4 College of Life Science, Shandong Agricultural University, Tai’an, 271018, China

**Keywords:** oomycetes, late blight, effector, *Solanum tuberosum*, growth inhibition, RNA-Seq

## Abstract

*Phytophthora infestans* causes the severe late blight disease of potato. During its infection process, *P. infestans* delivers hundreds of RXLR (Arg-x-Leu-Arg, x behalf of any one amino acid) effectors to manipulate processes in its hosts, creating a suitable environment for invasion and proliferation. Several effectors interact with host proteins to suppress host immunity and inhibit plant growth. However, little is known about how *P. infestans* regulates the host transcriptome. Here, we identified an RXLR effector, *PITG_15718.2*, which is upregulated and maintains a high expression level throughout the infection. Stable transgenic potato (*Solanum tuberosum*) lines expressing *PITG_15718.2* show enhanced leaf colonization by *P. infestans* and reduced vegetative growth. We further investigated the transcriptional changes between three *PITG_15718.2* transgenic lines and the wild type Désirée by using RNA sequencing (RNA-Seq). Compared with Désirée, 190 differentially expressed genes (DEGs) were identified, including 158 upregulated genes and 32 downregulated genes in *PITG_15718.2* transgenic lines. Eight upregulated and nine downregulated DEGs were validated by real-time RT-PCR, which showed a high correlation with the expression level identified by RNA-Seq. These DEGs will help to explore the mechanism of PITG_15718.2-mediated immunity and growth inhibition in the future.

## 1. Introduction

Plants have developed sophisticated surveillance systems to respond to pathogens and mount defenses against attack. When plants are constantly challenged by microbial parasites in the environment, they can defend themselves from most of the attacks through the innate immune system. Plants detect a multitude of potential invaders, including bacteria, fungi, and oomycetes and have evolved into a two-layered immunity system to protect themselves [1]. The pattern-triggered immunity (PTI) recognizes the microbe-associated molecular patterns (MAMPs) to trigger defense responses that can effectively defeat the vast majority of potential microbes. However, the pathogens secrete and deliver effectors into the host cells to directly manipulate the functions of immune regulators [2,3]. In turn, resistance proteins (R) recognize effectors, activating a rapid immune response known as effector-triggered immunity (ETI), which frequently results in a localized cell death known as the hypersensitive response (HR). Furthermore, effectors can evolve new functions through mutation to suppress ETI and promote disease progression [1,2].

*Phytophthora infestans* is a major threat to global food security, causing recurrent epidemics in the world’s fourth most important crop, potato [4]. However, the molecular interaction of this pathogen with plant hosts is poorly understood. To manipulate its hosts, *P. infestans* secretes effector proteins, some of which translocate into host cells [5,6,7,8]. Effectors may act outside or within plant cells to suppress immunity or modify other host processes to increase disease potential [9,10]. RXLR (Arg-x-Leu-Arg, x behalf of any one amino acid) effectors are the largest class of secreted proteins that are translocated into hosts [11,12]. Research on host targets of RXLR effectors has not only revealed essential virulence strategies of *Phytophthora* but also helped to identify novel components of plant immunity. The delivery of RXLR effectors inside plant cells can affect both plant PTI and ETI. Interestingly, due to the arms race between hosts and *Phytophthora*, RXLR effectors often act as virulence factors or avirulence factors, whether involved in PTI or ETI. For instance, the expression of *PexRD2* and *PITG_22798*, two RXLR effectors of *P. infestans*, induce HR in *Nicotiana benthamiana* [13,14]. In addition, 11 out of 169 RXLR effectors in *P. sojae* can induce HR in *N. benthamiana* [15]. They act as elicitors to trigger the host PTI. In contrast, many RXLR effectors inside plant cells can inhibit the HR induced by MAMPs to attenuate plant PTI. Eight out of thirty-three PiRXLR effectors were identified to suppress the early Flg22-induced immune response [16]. Infestin 1 (INF1) is the best-recognized MAMP, which triggers the HR in *N. benthamiana* [17]. However, Avr3a can suppress the INF1-triggered HR by targeting the E3 ligase CMPG1 in the host [17,18]. PexRD54 binds the host autophagy protein ATG8CL to interfere with Joka2-mediated defense in the host [19]. *Pi17316* also attenuates the INF1-triggered HR by targeting a MEK kinase (MAP3K) [20].

Alternatively, RXLR effectors play distinct roles in ETI. Currently, all known avirulence genes of potato late blight are RXLR effectors, such as Avr1, Avr2, AVR3a, Avr3b, Avr10, Avr11 and AVRblb1, which are recognized by cognate resistance proteins R1, R2/Rpi-blb3/R2-like, R3a, R3b, R10, R11 and Rpi-blb1. In addition, RXLR effectors ipiO1 and ipiO2 are recognized by RB [21,22,23,24,25,26,27]. On the other hand, some effectors can inhibit the HR triggered by the recognition of resistance proteins and the products of avirulence genes. PexRD2 directly targets and inhibits the MAPK3Kε activity and suppresses the HR mediated by the recognition of Avr4 by Cf4 [28]. Like PexRD2, Avr3a can suppress the HR triggered by the recognition of Avr4 by Cf4 [29]. Coinfiltration with Avr3b suppresses the PITG_22798 induced HR in *N. benthamiana* [16]. Wang et al. reported that many RXLR effectors of *P. sojae* inhibited the HR induced by Avh238 and Avh241 in *N. benthamiana* [17]. Meanwhile, successful pathogen effectors can escape the detection of resistance genes. *P. infestans* AVR3a^K80I103^ is recognized by the potato R3a protein. However, the allelic variant AVR3a^E80M103^ failed to be recognized by R3a [14]. Similarly, the amino acid substitution of AVRblb2 at position 69 was evolved to escape the recognization by its cognated Rpi-blb2 [15].

Moreover, to suppress the host immunity, pathogenic effectors can also alter plant development. By targeting BAK1 involved in signaling triggered by brassinosteroid (BR), multiple MAMPs and cell death, *Pseudomonas syringae* effectors of AvrPto and AvrPtoB suppress the plant immunity and development [30]. Overexpression of *Phytoplasma* effectors, SAP11, SAP54 and TENGU, lead to witches’ broom symptoms as well as enhance the susceptibility in Arabidopsis [31,32,33]. Transgenic plants carried the *Ralstonia solanacearum* effectors; RipAB exhibited a stunted growth in potato [34]. *Phytophthora parasitica* RXLR effector, penetration-specific effector 1 (PSE1), which increases pathogen susceptibility and interferes with auxin physiology [35]. Among *P. infestans* RXLR effectors, transgenic potato carrying Avr2 and PSR1 exhibited both enhanced susceptibility and growth inhibition, respectively [36,37]. However, the RXLR effectors exhibiting the above double functions are still limited.

In this study, we focused on an RXLR effector, PITG_15718.2, secreted by *P. infestans*. We found that *PITG_15718.2* was upregulated during infection. Constitutive expression of *PITG_15718.2* in potato promoted *P. infestans* infection and inhibited plant growth, causing decreased plant height. To investigate the underlying mechanisms of these effects, we employed RNA-Seq to study the transcriptome dynamics of interactions in *PITG_15718.2*-transformed lines and wild type. We identified 190 DEGs that were associated with the attenuated immunity and growth in *PITG_15718.2*-expressing potato.

## 2. Results

### 2.1. High Accumulation of the PITG_15718.2 Transcripts during P. infestans Infection

**Figure 1:**
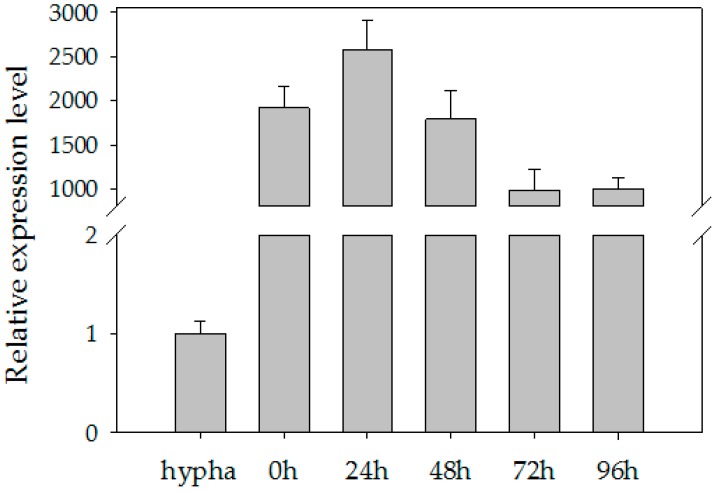
*PITG_15718.2* is upregulated in sporangia and during the early stages of infection. The hyphae were scraped with a glass rod from the plants on which *P. infestans* grew at 18 °C for two weeks. The sporangia were collected from the suspension, then incubated on ice and stored at 4 °C for 3 h to release the zoospores. The *PITG_15718* expression level was assessed by qRT-PCR at 0, 12, 24, 48, 72 and 96 h after inoculation of *P. infestans*. Error bars represent SD of the mean values of three biological replicates. The experiment was repeated three times with similar results.

Many RXLR effectors of *P. infestans* are upregulated during the biotrophic phase of infection on potato plants [20]. The gene *PITG_15718.2* is annotated as encoding a secreted RXLR effector protein in the *P. infestans* genome [11]. To investigate the expression of *PITG_15718.2* in *P. infestans* during infection, total RNA from hyphae and potato leaves at 0, 24, 48, 72 hand 96 h post-inoculation (hpi) were purified and used for real-time RT-PCR. The expression of *PITG_15718.2* was compared between lines after normalizing to the PI-O8, a repetitive element from *P. infestans* [38]. As shown in Figure 1, compared to hyphae (cultured nonsporulating mycelium), the expression of *PITG_15718.2* was rapidly increased to over 1900-fold at 0 h, which was represented as the sporangium and zoospore attached to plant tissue. It reached the highest expression level at 24 hpi. Three repeated amplifications in independent experiments with different cDNA samples resulted in similar expression patterns, confirming that *PITG_15718.2* is highly expressed during infection.

### 2.2. The Constitutive Expression of PITG_15718.2 in S. tuberosum

**Figure 2:**
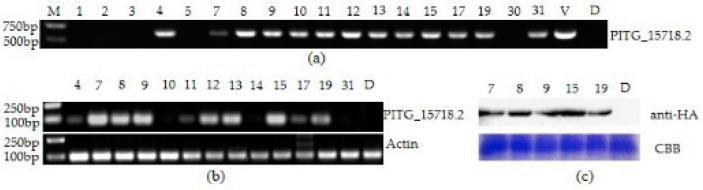
Validation of positive transgenic lines of *PITG_15718.2* potato (Désirée). (**a**) PCR detected 18 transgenic individuals using *PITG_15718.2*-specific primers with agarose gel electrophoresis. A total of 13 positive lines out of 18 lines were obtained; (**b**) detection of the expression level of *PITG_15718.2* using semiquantitative RT-PCR for 13 positive lines. The *StEF* gene was the endogenous control. *PITG_15718.2* was highly expressed in lines 7, 8, 9 and 15; (**c**) western blot showing that PITG_15718.2 was highly accumulated in lines 7, 8, 9 and 15. Total protein extracts from *S. tuberosum* (Désirée and lines 7, 8, 9 and 15). HA-tagged fusion proteins were detected by probing western blots with HA antibodies.

RXLR effectors often promote *P. infestans* virulence with specifically upregulated expression during the biotrophic phase of infection [39,40]. Désirée is a susceptible variety that is used for studying late blight disease resistance. To investigate the virulence function of *PITG_15718.2* during *P. infestans* infection, we transformed it into potato cultivar Désirée using an *Agrobacterium tumefaciens*-mediated system. In brief, a construct carrying the *PITG_15718.2* was cloned, with both a HA-tag and a CFP-tag fused to its N-terminus. A total of 18 transgenic plants were generated, and their DNA was extracted for positive selection. After PCR with specific primers, thirteen lines, numbered 4, 7, 8, 9, 10, 11, 12, 13, 14, 15, 17, 19 and 31, were identified to carry *PITG_15718.2* (Figure 2a). To investigate the expression level of *PITG_15718.2* in transgenic lines, the above thirteen positive lines were sampled to amplify the *PITG_15718.2* with semiquantitative RT-PCR. The results showed that the expression of *PITG_15718.2* was significantly higher in lines 7, 8, 9, 12, 13, 15 and 19 than the other lines (Figure 2b). Furthermore, lines 7, 8, 9, 15 and 19 highly expressed the PITG_15718.2 protein by western blot (Figure 2c). Thus, three transgenic lines, line 7, line 9 and line 15, were selected for further investigations.

### 2.3. PITG_15718.2 Promotes P. infestans Virulence in Potato

**Figure 3:**
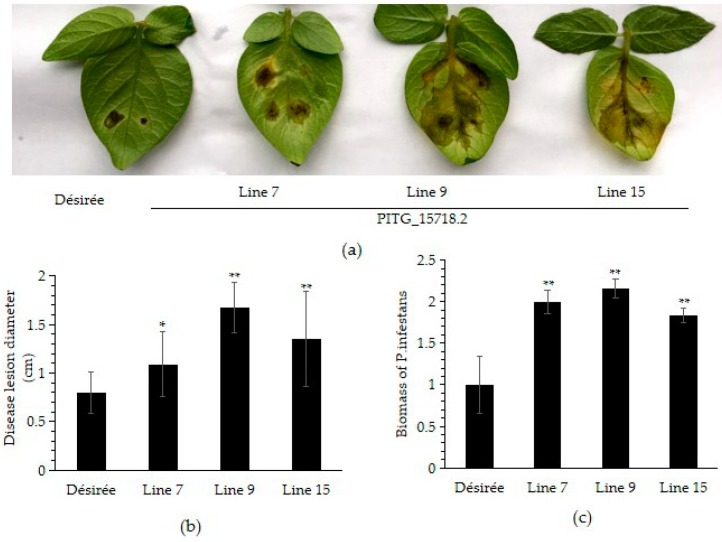
Constitutive expression of *PITG_15718.2* enhanced susceptibility to *P. infestans* in potato. (**a**) Three *PITG_15718.2*-expressing lines were challenged with *P. infestans* HLJ. Images were photographed at four days post-inoculation (dpi). Désirée was used as control; (**b**) the lesion size was significantly enhanced on both transgenic potato lines compared to the control. The disease lesion diameter was calculated from at least 15 leaves using the average of the longest and shortest diameters on each leaf. * indicates significant (*t* test, *p* < 0.05) differences, ** indicates extremely significant (*t* test, *p* < 0.01) differences; (**c**) determination of *P. infestans* biomass by quantitative real-time PCR. *P. infestans*–specific primers referred the PI-O8 element were used for PCR with the mixed-DNA template that was isolated from infected leaves (containing the DNA of *P. infestans* and *S. tuberosum*). The host gene (*StEF*) was used to normalize the results. ** indicates extremely significant (*t* test, *p* < 0.01) differences. The experiment was repeated three times with similar results.

To evaluate the virulence of *PITG_15718.2*, detached leaves of transgenic lines 7, 9 and 15 were inoculated with *P. infestans* HLJ at the 8-leaf stage. As shown in Figure 3a, images were photographed at 4 dpi. Compared to Désirée, the transgenic lines were more susceptible to *P. infestans* HLJ. Consistent with the greater susceptibility, the average leaf disease lesion diameter was longer on all three transgenic lines than Désirée (Figure 3b). Furthermore, using the mixed DNA from potato leaves and propagated *P. infestans* hyphae as template, we quantified the PI-O8 element with relative quantitative PCR, which represented the relative biomass of *P. infestans*. All three transgenic lines showed significantly increased relative biomass of *P. infestans* over Désirée (Figure 3c). These results demonstrated that the transgenic lines had enhanced *P. infestans* colonization and propagation compared with the wild type. This suggests that *PITG_15718.2* promotes *P. infestans* virulence in potato.

### 2.4. PITG_15718.2 Inhibits Potato Plant Growth

**Figure 4:**
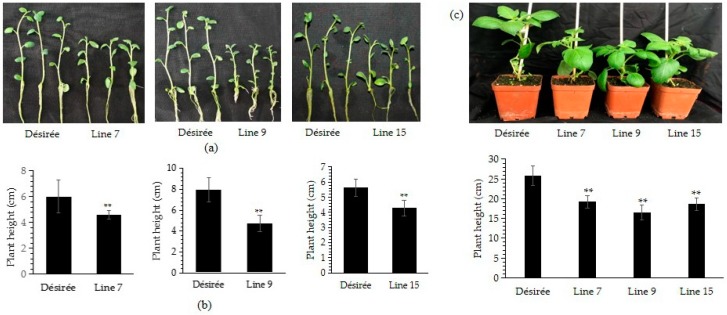
Stable expression of *PITG_15718.2* inhibits the vegetative growth of *S. tuberosum*. (**a**) Images of transgenic lines and controls. Each transgenic line was planted in one tissue-cultural container with Désirée. Three seedlings on each side, forming a confrontation. Images were photographed two weeks after planting; (**b**) the plant height was significantly dwarfed for each transgenic potato line compared to Désirée. The plant height was measured in at least 30 plantlets. ** indicates extremely significant differences determined using the Student’s *t*-test (*p* < 0.01); (**c**) the transgenic lines show stunted phenotype after planting in soil. Image was photographed three weeks after planting into soil. The plant height was measured in at least 20 plantlets. ** indicates extremely significant differences determined using the Student’s *t*-test (*p* < 0.01).

During the propagation of the tissue culture plantlets, we found that all transgenic plantlets were smaller than Désirée. Therefore, we cultured the plantlets of transgenic lines and wild type side by side in the same tissue-cultural container. After two weeks, plantlets from all three transgenic lines were smaller than Désirée (Figure 4a). The plant height was significantly shorter in the transgenic lines than in Désirée as well as the root length after measuring over 30 individuals from 10 bottles (Figure 4b and Figure S1). Furthermore, transgenic plants were exhibited the stunted phenotype three weeks after planting into soil (Figure 4c). So we concluded that the expression of *PITG_15718.2* inhibits the plant growth of Désirée.

### 2.5. Transcriptional Profiling Analysis of the Transgenic Lines Constitutively Expressing PITG_15718.2

**Figure 5:**
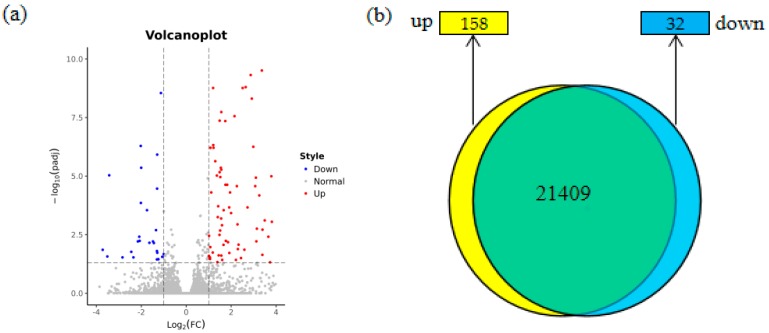
Differentially expressed genes in transgenic lines expressing *PITG_15718.2* compared with the wild type. (**a**) Volcano plot showing fold change and adjusted *p*-value of normalized read counts of the RNA-Seq data. The criteria of log_2_|(fold change)| ≥ 1 and padj ≤ 0.05 were used to identify the differentially expressed genes (DEGs). Blue dots indicate the downregulated DEGs (32 genes), and red dots indicate the upregulated DEGs (158 genes); (**b**) Venn diagram showing the overlap of the DEGs between the transgenic and wild type groups. The sum of all the numbers in the figure represents the total number of genes in the comparison. The overlapping region indicates the number of genes that were not significantly different between the groups. The yellow label indicates the number of upregulated DEGs and the cyan label indicates the number of downregulated DEGs according the set thresholds.

To explore the mechanism by which PITG_15718.2 suppressed potato immunity and vegetative growth, we compared the transcriptome profile of the three transgenic lines with their respective wild type controls by RNA sequencing. Six libraries were constructed and sequenced by an Illumina PE150. An average of 7 G Cleandata were obtained from each library, with over 97% of reads longer than 20 base pairs (bp) and approximately 43% G+C content (Table S1). The transcriptome sequencing reads were aligned to the potato DM genome [41] using the Hierarchical Indexing for Spliced Alignment of Transcripts (HISAT2) software. In summary, the number of RNA-Seq reads per library ranged from 49.0 to 63.9 million, in which 76.4% to 77.7% of reads mapped to the potato genome (Table S2). Then, we compared and analyzed the RNA-Seq data for the three transgenic lines and their wild type controls. We use HTSeq to calculate Reads Count and DEseq2 to compute differential expression. By defining a log_2_(fold change) ≥1 and adjusted *p*-value ≤0.05 as the standards of a differentially expressed gene (DEG), we identified 190 genes (Figure 5a), including 158 upregulated genes and 32 downregulated genes (Figure 5b), common to all three transgenic lines compared to Dall.

### 2.6. Functional Enrichment of the Differentially Expressed Genes

The functions of DEGs were associated with the phenotypes of immunity and growth inhibition in *PITG_15718.2*-expressing potato. To understand the biological functions of the DEGs, we carried out gene ontology (GO) analysis with the Hypergeometric distribution algorithm of Cluster Profiler [8] for the above 190 identified DEGs a *p* value less than 0.05. Among the 158-upregulated genes, their encoding proteins were classified into 22 GO enrichment summarized to three main categories including biological process, molecular function and cellular component (Table 1). For the proteins encoded by the 32-downregulated genes, 17 GO enrichments were classified and integrated into the three categories too (Table 2). Among the total of 39 GO enrichments, 35 enrichments were concentrated in biological process and molecular function. Among the upregulated DEGs, the GO term of the oxidation-reduction process, belonging to the enrichment biological process, largely overlapped with catalytic activity, belonging to molecular function. Among the downregulated DEGs, two GO terms of the biological process, response to external stimulus and symbiosis, encompassing mutualism through parasitism, were shared with most genes belonging to catalytic activity of molecular function. Whether upregulated or downregulated, the GO term of catalytic activity contained the highest number of proteins among all enrichments, indicating that they may not directly determine the PITG_15718.2 function (Table 1 and Table 2). Alternatively, we noticed that the functions were completely different under the biological process and molecular function between upregulated and downregulated proteins. The enrichment of the molecular function was concentrated on binding activity and lyase activity among upregulated and downregulated DEGs, respectively. Under biological process, the upregulated DEGs were mainly distributed into porphyrin-containing compound biosynthetic process (GO:0006779), cell differentiation (GO:0030154), cofactor biosynthetic process (GO:0051188), multiorganism process (GO:0051704), pigment biosynthetic process (GO:0046148) and oxidation-reduction process (GO:0055114). However, the biological processes of downregulated DEGs contained the enrichments of response to external stimulus (GO:0009605), response to other organism (GO:0051707), response to external biotic stimulus (GO:0043207), symbiosis, encompassing mutualism through parasitism (GO:0044403), interspecies interaction between organisms (GO:0044419) and response to biotic stimulus (GO:0009607). Those functions are clearly related to plant immunity, which may help to explain the virulence function of PITG_15718.2.

### 2.7. Validation of DEGs by Real-Time RT-PCR

**Figure 6:**
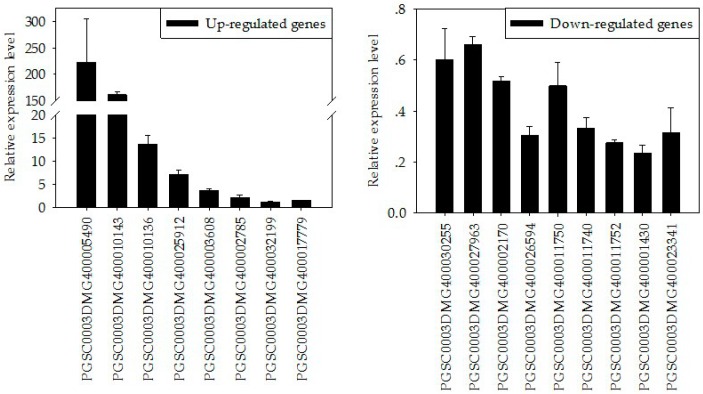
Validating the DEGs by qRT-PCR. (**a**) Eight of the upregulated DEGs; (**b**) nine of the downregulated DEGs. Error bars represent SD of the mean of three biological replicates. The experiment was repeated three times with similar results.

To validate the RNA-Seq results, we selected several DEGs to perform the real-time RT-PCR detection. Based on their putative functions in plant immunity or growth regulation, eight of the upregulated and nine of the downregulated genes were selected. As shown in Figure 6a,b, all seventeen genes showed differential expression in transgenic lines. Compared with the RNA-Seq fold-change numbers, except PGSC0003DMG400032199, the other 16 DEGs showed extremely tight correlations with the qRT-PCR results (Figure S2).

### 2.8. Expression of PITG_15718.2 Decrease the Content of Indole-3-Acetic Acid

**Figure 7:**
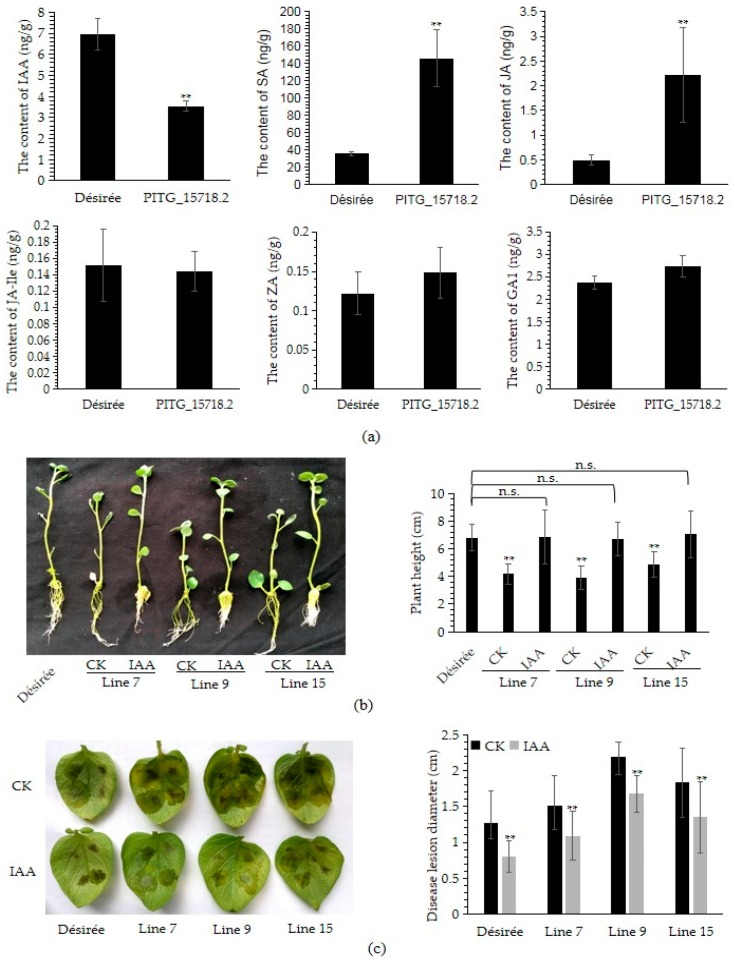
Decreasing of IAA is involved in PITG_15718.2-mediated inhibition of plant immunity and growth. (**a**) The content of salicylic acid (SA), jasmonic acid (JA), Ile-JA, Zeatin (ZA), indole-3-acetic acid (IAA) and gibberellic acid 1 (GA1). The sample was collected from leaves of 3-weeks of potato planting in soil. The sample of PITG_15718.2 was mixed with equal amount of three transgenic lines 7, 9 and 15. Four replicates for each sample were measured by LC-MS. Asterisks indicate extremely significant differences determined using the Student’s *t*-test (*p* < 0.01); (**b**) the plant height of transgenic lines is restored by supplementing with IAA. Images were photographed two weeks after planting. The plant height was measured with at least 20 plantlets. The concentration of IAA is 1 mg/L; (**c**) pre-spraying IAA enhances resistance to *P. infestans.* Leaves of 3-weeks of potato planting in soil was sprayed with 1 mg/L of IAA and inoculated with *P. infestans* 24 h later. Images were photographed four days post inoculation and disease lesion diameter were measured with at least 30 leaves. Asterisks indicate extremely significant differences determined using the Student’s *t*-test (*p* < 0.01). The above experiments (**b**,**c**) were repeated three times with the similar results.

The expression of *PITG_15718.2* increases the susceptibility to *P. infestans* and reduces the plant growth, indicating that phytohormones may be involved in the signaling pathway. Therefore, we quantify the content of salicylic acid (SA), jasmonic acid (JA), Ile-JA, Zeatin (ZA), indole-3-acetic acid (IAA) and gibberellic acid 1 (GA1) by liquid chromatograph-mass spectrometer (LC-MS) for both Désirée and mixed samples of three transgenic lines. As shown in Figure 7a, compared to Désirée, the content of IAA was dramatically decreased in transgenic lines while the content of SA and JA were increased. There were no significant changes for the content of Ile-JA, ZA and GA1 between Désirée and mixed transgenic samples. The high content of SA and JA were associated to enhance the potato resistance to *P. infestans*, and IAA was associated with promoting plant growth. So we select IAA for the growth restoration assays with *PITG_15718.2* transgenic lines. As shown in Figure 7b, supplementing with 1 mg/L of IAA, all three transgenic lines can restore the plant height as well as wild type Désirée. In addition, we found that the IAA-treated plantlets show more numbers but shorter adventitious roots than untreated controls. Furthermore, we found that pre-sprayed IAA could enhance the resistance to *P. infestans* for both Désirée and transgenic plants (Figure 7c). Overall, the results were implied that PITG_15718.2 may suppress the plant immunity and reduce growth by decreasing the content of IAA.

## 3. Discussion

*P. infestans* is a particularly destructive pathogen causing late blight that leads to significant potato crop losses due to early defoliation and tuber infection. The pathogen spreads rapidly, causing complete crop loss within days without successful management [42]. RXLR effectors are secreted by *P. infestans* to manipulate the host’s physiology to promote infection, including debilitation of the immune system. The genome sequence shows that there are more than 500 RXLR effectors in *P. infestans*. The RXLR effectors promoting the infection of *P. infestans* are upregulated during the infection stage [39,40]. Assays of upregulated RXLRs have indicated that 45 out of 52 RXLRs promote pathogen leaf colonization when expressed in *N. benthamiana* [43]. Nevertheless, for the majority of RXLR effectors, their biological functions and potential host targets are unknown. In this study, we identified that the RXLR effector encoded by *PITG_15718.2* is upregulated during the infection stage of *P. infestans* and maintains a high expression throughout the infection phase. Consistent with previous reports, constitutive expression of *PITG_15718.2* increased the susceptibility of potato to *P. infestans* in detached leaves. We conclude that *PITG_15718.2* acts as a virulence factor in the interaction of *P. infestans* and *S. tuberosum*, providing another example of an RXLR effector as a virulence factor.

Most identified RXLR effectors of *P. infestans* are involved in regulating plant immunity, whether directly as virulence factors as discussed above, or indirectly as avirulence factors recognized by their cognate R proteins. As virulence factors, knowledge of their functions in regulating plant growth are still limited. Phytohormones, such as SA, JA and IAA, broadly involved in regulating both plant immunity and growth. SA antagonize JA and repress auxin accumulation in plant defense. However, the crosstalk of phytohormones mediated by the RXLR effector is poorly understood. Constitutively expressed Avr2, an RXLR effector, interferes with plant growth through overactive brassinosteroid (BR) hormone signaling [36]. Overexpression of *StPSR1* could inhibit the growth of Arabidopsis, and the root hairs phenotype could significantly decrease about 80% after IAA treatment [37]. Here, we showed that expressing *PITG_15718.2* also significantly decreased plant height (Figure 4a) and the content of IAA (Figure 7a). IAA treatment could restore the plantlets height and increase the disease resistance to *P. infestans* (Figure 7b,c). It was demonstrated that both the PITG_15718.2-mediated susceptibility and growth inhibition were partially caused by decreasing of the IAA content. It is similar as the *Phytoplasma* effector TENGU that promoted the infection and led to dwarfism by downregulating the auxin-related genes [31]. Meanwhile, we also have noticed the accumulation of higher content of SA in transgenic plants than in Désirée (Figure 7a). High accumulation of SA would reduce the IAA accumulation and cause the inhibition of plant growth. Additionally treatment with SA has been previously reported to increase the resistance or susceptibility to *P. infestans* on susceptible potato [44]. Whether and how the PITG_15718.2 regulates the SA and IAA in Désirée needs to be further studied.

Next-generation sequencing (NGS) technologies are fast evolving and transforming biology research [45]. RNA-Seq is an approach to study the transcriptome [46,47]. RNA-Seq is a valuable method for transcriptome dynamics analysis in tetraploid potato [48,49,50]. This suggests that RNA-Seq could be used to study potato immunity and growth. However, to understand the function of RXLR effectors, previous studies mainly used the protein–proteininteraction system to identify their host targets, as well as to determine the biological functions in regulating host immunity. For instance, Avr3a modifies the E3 ubiquitin ligase CMPG1, resulting in accumulation of CMPG1 and making plants more susceptible [14]. Quite coincidentally, to weaken the plant immunity, the Pi02860 target host NRL1, a BTB-domain protein that forms the substrate adaptor component of a CULLIN3 ubiquitin E3 ligase, degrades SWAP70, a positive regulator of immunity [51]. PexRD2 targets MAPKKKε to suppress host immunity [28]. Avrblb2 interacts with the cysteine caspase C14 to prevent C14 secretion and destroy host immunity [52]. PexRD54 is able to combine with ATG8 to interfere with the host selective autophagy [19]. However, after these protein–protein interactions, the downstream signaling transduction pathways are still unknown. Recently, by analyzing BR-treatment microarray data, a BR-inducible bHLH transcription factor, StCHL1, was identified to be upregulated by AVR2 and to be required for its suppression of INF1-mediated HR [36]. To explore the mechanism by which PITG_15718.2 affects plant immunity and growth, we performed the RNA-Seq analysis of transgenic plants. We conducted the RNA-Seq on three independent transgenic lines in parallel to eliminate the effects caused by different T-DNA insertions. Only 190 DEGs, including 158 upregulated and 32 downregulated, were identified.

Interestingly, among upregulated DEGs, *PGSC0003DMG400003608*, encoding an abscisic acid 8’-hydroxylase, is reminiscent of a previous finding of negatively regulated plant growth because overexpression of the homologue of *GhABAH* was found to inhibit the growth in transgenic tomatoes and cotton [53]. Overexpression of ShMKS1 in *Arabidopsis thaliana* and *Nicotiana tabacum* also caused serious growth defects [54]. The upregulated DEG *PGSC0003DMG400025912* is a homologue of ShMKS, which encodes a salicylic acid-binding protein and may negatively regulate plant growth. *PGSC0003DMG400010143* encodes a cysteine protease inhibitor gene, which was upregulated during *P. infestans* infection of tomato, suggesting a role during *P. infestans*-host interactions [55]. These data indicate that the *PITG_15718.2*-induced DEGs may negatively regulate the plant immunity or vegetative growth. In contrast to upregulated DEGs, among downregulated genes, cytochrome P450 (*PGSC0003DMG400026594* and *PGSC0003DMG400011750*) are essential for lignification and defense against predators and pathogens [56]. The transgenic potato carrying the antisense of Gibberellin 20 oxidase (*PGSC0003DMG400027963*) cDNA had a shorter stem, a decreased length of the internodes and tuberized earlier than control plants, showing increased tuber yields [57]. Mutants at the PROCUSTE1 (PRC1) or RSW1 (CesA1) loci (cellulose synthase, *PGSC0003DMG400011752*) showed decreased cell elongation, specifically in roots and dark-grown hypocotyls [58]. *PGSC0003DMG*400030255 encodes a member of YUCCA gene family, which is an important regulator of IAA biosynthesis. Consistent with its downregulated expression, the content of IAA is dramatically reduced in transgenic lines than in Désirée (Figure 7a). The above cases suggest that the downregulated DEGs in *PITG_15718.2* transgenic potato may positively regulate immunity and plant growth. They are likely to be key components downstream of the interaction between PITG_15718.2 and host targets that need to be investigated in the future.

## 4. Materials and Methods

### 4.1. Microorganism and Plant Materials

*Escherichia coli* DH5a and *Agrobacterium tumefaciens* GV3101 and AGL1were routinely grown in Luria–Bertani (LB: 10 g of bacteriological peptone, 10 g NaCl, and 5 g yeast extract in 1 L of distilled water) medium plus with appropriate antibiotics at 37 and 28 °C, respectively. *P. infestans* strain HLJ, originally isolated from the province of Heilongjiang, north of China, was grown in the dark at 18 °C using rye A agar. Unless noted, *S. tuberosum* and *N. benthamiana* plants were grown at 22 and 25 °C, respectively, under 70% humidity with 16 h of light and 8 h of dark.

### 4.2. Construction of Recombinant Binary Vector

Since none of the tested *Avr* genes contained predicted introns, we amplified each of the genes directly from genomic DNA from the *P. infestans* HLJ, using the Cobuddy DNA Polymerase (CWBIO, Beijing, China) and specific primers. The PCR products were digested with the appropriate enzymes and cloned into the recombinant binary vector pEarleyGate 102 (C-CFP-HA) [59] and PVX vector pGR106. The constructs were validated by DNA sequencing then introduced into *Agrobacterium tumefaciens* GV3101 or AGL1.

### 4.3. Transformation of Potato

*Agrobacterium tumefaciens* AGL1 containing pEarleyGate 102-PITG_15718.2 was used for transformation with the stem of the potato cv. Désirée as previously reported [60]. Positive lines were first screened on differential medium (MS + 0.2 mg·L^−1^ NAA + 0.02 mg·L^−1^ GA3 + 2.5 mg·L^−1^ zeatin riboside + 500 mg·L^−1^ cefotaxime) and then transferred to root generation medium (MS + 0.02 mg·L^−1^ NAA + 0.02 mg·L^−1^ GA3 + 2 mg·L^−1^ zeatin riboside + 500 mg·L^−1^ cefotaxime + 50 mg/L glufosinate). The positive selection and the expression level of *PITG_15718.2* was investigated with PCR, semiquantitative RT-PCR and western blot.

### 4.4. Gene Expression Analysis by qRT-PCR

Total RNA was isolated with 100 mg fresh tissue using the TRI Reagent (Merck, Darmstadt, Germany). The residual DNA removal and first-strand cDNA synthesis were conducted with ReverTra Ace qPCR RT Master Mix with gDNA Remover kit (TOYOBO, Osaka, Japan). The real-time RT-PCR was done with KOD SYBR qPCR Mix (TOYOBO, Osaka, Japan). For PCR, samples were preheated at 95 °C for 5 min. Then, 40 amplification cycles were run: 15 s at 95 °C, 30 s at 60 °C. Fluorescence (520 nm) was detected at the end of the elongation phase for each cycle. After the final amplification cycle, a melting curve was done by heating to 95 °C, cooling to 65 °C and slowly heating to 95 °C at 0.1 °C·s^−1^ with continuous measurement of fluorescence at 520 nm.

### 4.5. Detached Leaf Assays

*P. infestans* HLJ was grown in petri dishes in Rye A for two weeks at 18 °C. Plates were flooded with 5 mL H_2_O and scraped with a glass rod to release sporangia. The suspension was poured into a clean Petri dish, placed on ice and stored in the 4 °C for 3 h to release zoospores. The sporangia were counted using a hemocytometer and adjusted to 30,000 sporangia per milliliter. The leaves were placed on moistened tissue paper in a 25-by-25-cm plate, then inoculated with 20 μL droplets of a zoospores and sporangia suspension. Inoculated leaves were incubated at 18 °C (16 h of light and 8 h of dark). The lesions were measured at 5 dpi. For IAA treatment, detached leaves were sprayed with the 1 mg/L of IAA and incubated for 24 h, then inoculated with *P. infestans*.

### 4.6. Determination of Pathogen Biomass by Quantitative Real-Time PCR

The biomass of *P. infestans* was determined using the modified method of Kobayashi [61]. To determine the growth of *P. infestans*, inoculated potato leaves were used for quantitative real-time PCR. Tissue was broken in liquid nitrogen. The DNA was isolated with the CTAB method. To amplify and detect *P. infestans* and plant specific DNA sequences [38], the following primers were used: O8-3 (5′-GAAAGGCATAGAAGGTAGA-3′) and O8-4 (5′-TAACCGACCAAGTAGTAAA-3′) for PI-O8 element of *P. infestans*; and StEF-1a -F(5′-GGTCTACCAACCTCGACTGGTAC-3′) and StEF-1a -R(5′-GGGTTTGTCTGATGGCCTCTTGG-3′) for potato *StEF* (AB061263) gene. The PCR system was as described above.

### 4.7. RNA-Seq

The plantlets of three *PITG_15718.2* transgenic lines and the wild type were planted side by side in the same tissue culture bottle for 20 days at 18 °C. Each bottle contained three plantlets for transgenic and three for Dhree f. There were at least five bottles for each experiment. For preparing each pooled RNA sample, a total of 15 seedling plants from five bottles were collected and submitted to total RNA extraction as described above. The quantity and quality of RNA samples were assessed using a NanoDrop One (Thermo Scientific, Waltham, MA, USA), Qubit 3.0 (Thermo Scientific) and 4200 TapeStation (Agilent, Palo Alto, CA, USA). High-quality total RNA (5 µg, 100 ng/μL) samples were sent to the HaploX Genomics Center (www.haplox.cn, shangrao, China) for RNA-Seq with an Illumina PE150.

### 4.8. Determination of the Phytohormones Concentration

The concentration of SA, IAA, JA, Ile-JA, ZA and GA1 was determined using the Shimadzu LCMS 8040 system (Shimadzu, Tokyo, Japan) as previously described [62]. Approximately 200 mg of leaf tissue was collected from the upper-full-expand leaves of 3-week-old wild type and ground in liquid nitrogen. The hormones were extracted with 1 mL of ethyl acetate spiked with each internal standard and quantified with LC-MS. Four replicates were conducted for each sample.

### 4.9. Deposition of Sequences

The data sets supporting the results of this article are included within the article and its additional files. The nucleic acid sequence of *PITG_15718.2* is available in NCBI’s database with accession number XM_002996840.1 (http://www.ncbi.nlm.nih.gov/nucleotide). The RNA-Seq data supporting the results of this article are available in the NCBI’s SRA with the accession number SRR8392524 (http://www.ncbi.nlm.nih.gov/sra/).

## 5. Conclusions

In this study, we identified a novel RXLR effector of *P. infestans*, PITG_15718.2, a virulence factor that promoted the colonization of *P. infestans* and inhibited the vegetative growth of potato. We also identified 190 DEGs and the content of IAA that were associated with the attenuated immunity and growth in *PITG_15718.2*-expressing potato.

## Figures and Tables

**Table 1:** Gene ontology (GO) enrichments of upregulated genes.

GO_Accession	Description	DEG Number	Term Type
GO:0055114	oxidation-reduction process	12	biological process
GO:0051704	multi-organism process	7	
GO:0051188	cofactor biosynthetic process	3	
GO:0006779	porphyrin-containing compound biosynthetic process	2	
GO:0030154	cell differentiation	2	
GO:0046148	pigment biosynthetic process	2	
GO:0044427	chromosomal part	3	cellular component
GO:0032040	small-subunit processome	2	
GO:0030684	Preribosome	2	
GO:0003824	catalytic activity	42	molecular function
GO:1901363	heterocyclic compound binding	36	
GO:0097159	organic cyclic compound binding	36	
GO:0043168	anion binding	17	
GO:0036094	small molecule binding	17	
GO:0000166	nucleotide binding	16	
GO:1901265	nucleoside phosphate binding	16	
GO:0003677	DNA binding	13	
GO:0020037	heme binding	6	
GO:0046906	tetrapyrrole binding	6	
GO:0016705	oxidoreductase activity, acting on paired donors, with incorporation or reduction of molecular oxygen	5	
GO:0004674	protein serine/threonine kinase activity	3	
GO:0045735	nutrient reservoir activity	2	

**Table 2:** GO enrichments of downregulated genes.

GO_Accession	Description	DEG Number	Term Type
GO:0009058	biosynthetic process	8	biological process
GO:0009605	response to external stimulus	3	
GO:0044403	symbiosis, encompassing mutualism through parasitism	3	
GO:0044419	interspecies interaction between organisms	3	
GO:0051704	multi-organism process	3	
GO:0006814	sodium ion transport	2	
GO:0051707	response to other organism	2	
GO:0043207	response to external biotic stimulus	2	
GO:0016051	carbohydrate biosynthetic process	2	
GO:0009607	response to biotic stimulus	2	
GO:0015672	monovalent inorganic cation transport	2	
GO:0044723	single-organism carbohydrate metabolic process	2	
GO:0005886	plasma membrane	2	cellular component
GO:0003824	catalytic activity	14	molecular function
GO:0016829	lyase activity	3	
GO:0016831	carboxy-lyase activity	2	
GO:0016830	carbon-carbon lyase activity	2	

## Supplementary Materials

Supplementary materials can be found at https://www.mdpi.com/1422-0067/20/12/3031/s1.

## Abbreviations

DEG	differentially expressed gene
ETI	effector-triggered immunity
HR	hypersensitive response
LD	linear dichroism
MAMPs	microbe-associated molecular patterns
PTI	pattern-triggered immunity
R	resistance protein
RNA-Seq	RNA sequencing
