# Supplementary material for: Transgenic RXLR Effector PITG_15718.2 Suppresses Immunity and Reduces Vegetative Growth in Potato
Source: Int J Mol Sci. 2019 Jun 21;20(12):3031. doi: 10.3390/ijms20123031 (PMC6627464; doi:10.3390/ijms20123031)
Supplement: Supplementary file 1 [file ijms-20-03031-s001.pdf]

Supporting informations

Table S1. Statistics of preprocessing data.

Sample	RawData(G)	CleanData(G)	Effective(%)	Q20(%)	Q30(%)	Error rate(%)	GC Content
CK15-1	7.61	7.31	96.15	97.47	93.63	0.01	42.97
CK7-1	9.9	9.1	95.99	97.44	93.57	0.01	43.14
CK9-1	8.15	7.79	95.55	97.37	93.42	0.01	42.7
15-1	8.51	8.15	95.78	97.4	93.49	0.01	43.17
7-1	7.67	7.3	95.17	97.28	93.25	0.01	42.8
9-1	8.14	7.77	95.47	97.38	93.47	0.01	42.64

(1). Sample: Sample name;

(2). RawData (G): The original data, in units of G;

(3). CleanData (G): The amount of valid data obtained by filtering, in units of G;

(4). Effective (%): representing the percentage of CleanData to RawData;

(5). Q20 and Q30 (%): The percentage of bases with Phred values greater than 20 and 30 was calculated respectively;

(6). Error rate (%): sample error rate;

(7). GC Content: Calculate the total number of bases G and C and the percentage of the total number of bases.

Table S2. The statistics of sequencing data is aligned to the reference genome

Sample	Total reads	Total mapped	Multiple mapped	Uniquely mapped	Non-splice reads	Splice reads
CK15-1	49052440	37546312 (76.5%)	3448723 (7.0%)	34097589 (69.5%)	21490194 (43.8%)	16056118 (32.7%)
CK7-1	63850896	49402912 (77.4%)	5538661 (8.7%)	43864251 (68.7%)	28753868 (45.1%)	20649044 (32.3%)
CK9-1	52271126	39942371 (76.9%)	3356865 (6.4%)	36585506 (70%)	22525996 (43.1%)	17416375 (33.3%)
15-1	52267810	40263471 (77%)	3426392 (6.5%)	36837079 (70.5%)	22687995 (43.4%)	17575476 (33.6%)
7-1	54765606	42534598 (77.7%)	4335471 (7.9%)	38199127 (69.8%)	24219309 (44.3%)	18315289 (33.4%)
9-1	49119334	38182638 (77.7%)	3768834 (7.6%)	34413804 (70.1%)	21728404 (44.2%)	16454234 (33.5%)

(1). Sample: sample name;

(2). Total reads: Valid data obtained by quality control of sequencing data (Clean data);

(3). Total mapped: Quantitative statistics of sequencing sequences that can be mapped to the genome;

(4). Multiple mapped: Quantitative statistics of sequencing sequences with multiple alignment positions on the reference sequence;

(5). Uniquely mapped: the number of sequencing sequences with unique alignment positions on the reference sequence;

(6). Non-splice reads: Complete comparison of sequenced Read statistics with exon regions;

(7). Splice reads: Segmentation alignment to the statistics of sequencing reads on two exons.

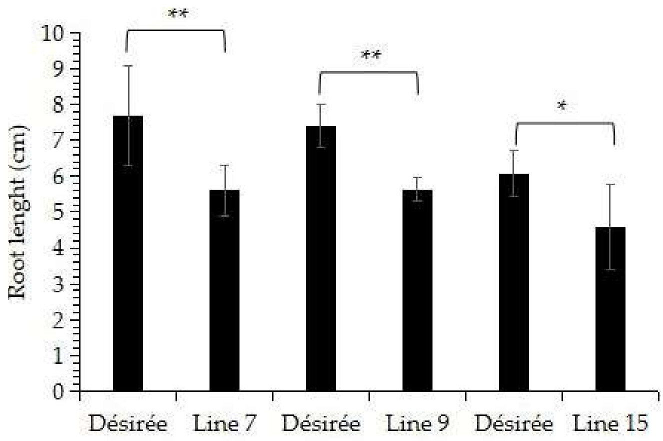

Figure S1. The root length was stunted for transgenic lines compared to Désirée. The root length was measured with at least 30 plantlets. "**" and "*" indicates significant differences determined using Student's *t*-test ($P < 0.01$ and $P < 0.05$) respectively.

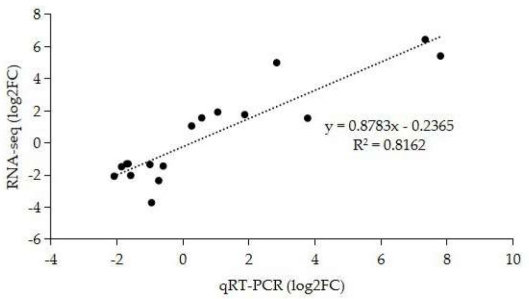

Figure S2. Correlation of the data of 17 DEGs between qRT-PCR and RNA-Seq. The pearson correlation coefficient reached 0.9014.
